# Supplementary figures and images for: Fast cyclic stimulus flashing modulates perception of bi-stable figure
Source: PeerJ. 2018 Nov 27;6:e6011. doi: 10.7717/peerj.6011 (PMC6266943; doi:10.7717/peerj.6011)

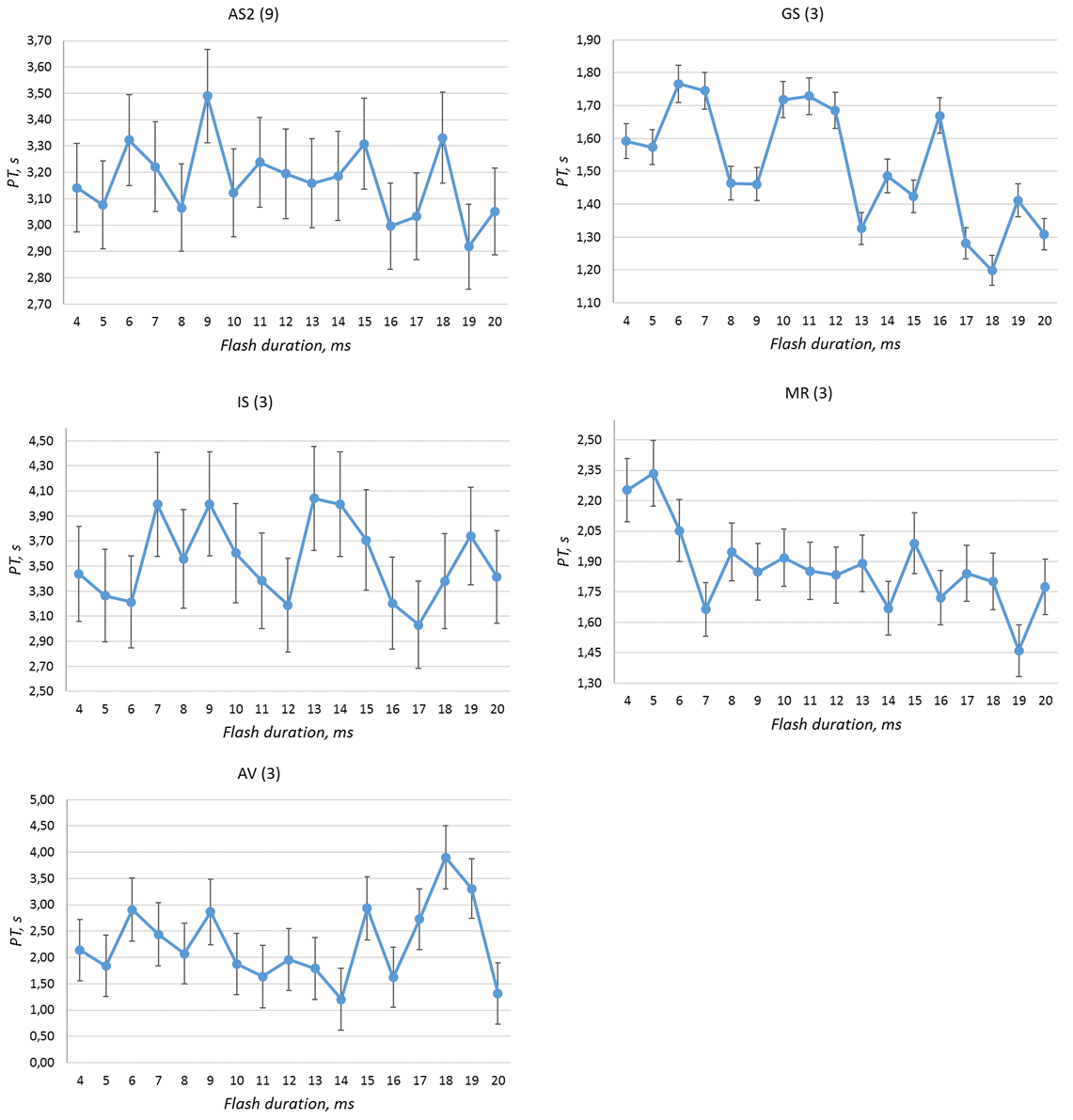

Supplement: Figure S1 — The abscissae—duration of flashing stimulus (ms), the ordinate—perception time value (s). Capital letters on the top of every picture mark different observers (number of all sessions, on which the data was collected, is in the brackets). Error bars represent the 95% confidence intervals (CI). [file peerj-06-6011-s002.png]
